# 3D Optical Imaging as a New Tool for the Objective Evaluation of Body Shape Changes After Bariatric Surgery

**DOI:** 10.1007/s11695-020-04408-4

**Published:** 2020-01-21

**Authors:** Andreas Kroh, Florian Peters, Patrick H. Alizai, Sophia Schmitz, Frank Hölzle, Ulf P. Neumann, Florian T. Ulmer, Ali Modabber

**Affiliations:** 1 Department of General, Visceral and Transplantation Surgery, RWTH Aachen University Hospital, Pauwelsstraße 30, 52074 Aachen, Germany; 2 Department of Oral, Maxillofacial and Plastic Facial Surgery, RWTH Aachen University Hospital, Pauwelsstraße 30, 52074 Aachen, Germany; 3 Department of Surgery, Maastricht University Medical Centre, P. Debyelaan 25, 6229 HX Maastricht, The Netherlands

**Keywords:** Bariatric surgery, Body shape, Three-dimensional imaging, Central obesity

## Abstract

**Introduction:**

Bariatric surgery is the most effective treatment option for obesity. It results in massive weight loss and improvement of obesity-related diseases. At the same time, it leads to a drastic change in body shape. These body shape changes are mainly measured by two-dimensional measurement methods, such as hip and waist circumference. These measurement methods suffer from significant measurement errors and poor reproducibility. Here, we present a three-dimensional measurement tool of the torso that can provide an objective and reproducible source for the detection of body shape changes after bariatric surgery.

**Material and Methods:**

In this study, 25 bariatric patients were scanned with Artec EVA®, an optical three-dimensional mobile scanner up to 1 week before and 6 months after surgery. Data were analyzed, and the volume of the torso, the abdominal circumference and distances between specific anatomical landmarks were calculated. The results of the processed three-dimensional measurements were compared with clinical data concerning weight loss and waist circumference.

**Results:**

The volume of the torso decreased after bariatric surgery. Loss of volume correlated strongly with weight loss 6 months after the operation (*r* = 0.6425, *p* = 0.0005). Weight loss and three-dimensional processed data correlated better (*r* = 0.6121, *p* = 0.0011) than weight loss and waist circumference measured with a measuring tape (*r* = 0.3148, *p* = 0.1254).

**Conclusion:**

Three-dimensional imaging provides an objective and reproducible source for the detection of body shape changes after bariatric surgery. We recommend its use for the evaluation of central obesity, particularly for research issues and body imaging before and after bariatric surgery.

**Electronic supplementary material:**

The online version of this article (10.1007/s11695-020-04408-4) contains supplementary material, which is available to authorized users.

## Introduction

Overweight and obesity are among the world’s largest health care problems, with more than 1.9 billion affected adults worldwide [1]. In addition to functional and esthetic limitations, obesity is associated with a large number of concomitant diseases that significantly reduce life expectancy [2].

Bariatric surgery can significantly reduce an individual’s weight. Within 6–12 months, an excess weight loss (%EWL) of 60–70% can be achieved [3, 4]. In addition to %EWL and the reduction of metabolic diseases, this massive weight loss also results in an enormous change in body shape. This is currently measured primarily by hip and waist circumference measurements. These two parameters are also used for diagnosing metabolic syndrome [5] or in various clinical scores such as the fatty liver index (FLI) for noninvasive diagnosis of nonalcoholic fatty liver disease (NAFLD) [6]. However, these two-dimensional measurement methods suffer from large measurement errors due to the lack of independent reproducibility and difficult anatomical conditions in obese patients with concealed anatomical landmarks and folds of sagging skin after bariatric surgery. Although the WHO has recommendations for measuring waist and hip circumference [7], especially in super obese patients, it is technically difficult to measure hip and waist circumference with a measuring tape in a reliable manner.

Three-dimensional imaging techniques using modern bedside 3D scanners are becoming increasingly popular in medicine. First, these scanners work without harmful ionizing radiation and are easy to use with a modern laptop. Furthermore, detailed three-dimensional information allows postprocessing and objective and reproducible data collection. These data are already used for evaluating patient outcome or planning complex surgical procedures in plastic reconstructive surgery or facial surgery [8–10].

The aim of the present study is to investigate three-dimensional measurements of the torsos of bariatric surgery patients with Artec EVA®, a state-of-the-art 3D scanner. Pre- and postoperative scans are matched and compared to visualize body shape changes. Postprocessed three-dimensional data are also compared and correlated with conventionally acquired data.

## Materials and Methods

### Study Design

After institutional approval and written informed consent, this pilot study was conducted in 25 patients to perform a preliminary investigation whether a three-dimensional measurement of the torso before and after metabolic surgery is feasible. All patients included in the study were assigned to metabolic surgery at the interdisciplinary bariatric center of the RWTH Aachen University Hospital between January 2016 and December 2017. Participants were bariatric surgery candidates with body mass indices of > 40 kg/m^2^ or > 35 kg/m^2^ with weight-related comorbidities and were assessed by a multidisciplinary team before surgery. Age < 18 years, epilepsy or any other seizure disorders triggered by bright or flashing lights were exclusion criteria. Three-dimensional imaging of the torso was conducted within 1 week before and 6 months after surgery. Clinical data (age, body weight, body height, and comorbidities) were pseudonymized and prospectively collected in the metabolic surgery database (DGAV-StuDoQ, Deutsche Gesellschaft für Allgemein- und Viszeralchirurgie e.V., Germany). The study was conducted in accordance with the 1964 Declaration of Helsinki and its later amendments and had received prior approval by the Independent Ethics Committee responsible for RWTH Aachen Faculty of Medicine under the reference number EK 174-19.

## Three-Dimensional Imaging

### Study Protocol

**Fig. 1:**
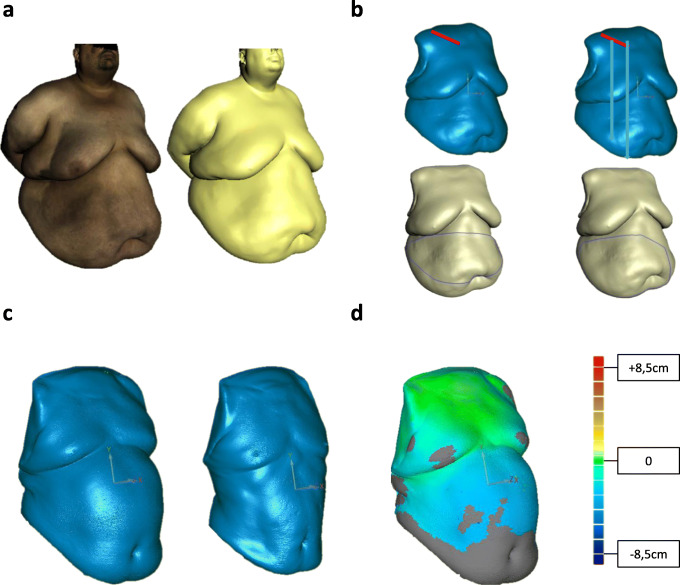
Three-dimensional reconstructions of the torso. **a** Three-dimensional scan before alignment with and without texture. **b** Three-dimensional reconstruction of the torso. The arms, legs, and head were removed for three-dimensional measurement. The clavicula is marked in red. Bright lines in blue torsos depict *d*_umb_ and *d*_es._ Purple lines in green torsos depict *c*_max_ and *c*_umb_. **c** Pre- and postoperative torso. **d** Heat map of matched pre- and postoperative scans depicting areas of body shape changes. The colored scale illustrates the extent of body shape change in centimeters. Gray color depicts areas of body shape change > 30 cm

All scans were performed with an Artec EVA® Scanner (Artec Group, Luxembourg, Luxembourg) following a standardized protocol. Participants stood straight with their feet close together and arms crossed behind their back to expose both claviculae (Fig. 1a). Men were scanned with their upper body undressed, and women were scanned in their bras.

### Artec EVA® and Structured Light Triangulation

Artec EVA® is a state-of-the-art technology for 3D surface scanning. It uses the structured light triangulation technique to receive 3D data from surfaces [11]. Two cameras take pictures of a certain point *P*. Predefined light patterns of parallel stripes are used to identify *P* in both pictures, and a three-dimensional picture is constructed.

Particularly important for daily use is the compact and easy-to-use nature of the application of the system and the lack of harmful radiation. To perform a 3D scan, Artec EVA® is circled around the object at a working distance of 40–100 cm until every spot of interest is captured. A picture and a video of performing the scan are included online as supplementary material. Data files were processed using Artec Studio software version 11.0 (Artec Group, Luxembourg, Luxembourg), and a 3D scan was created as a Joint Photographic Experts Group File Interchange Format (.jpg) (Fig. 1a) together with texture mapping information inside a Material Template Library file (.mtl). Artec Studio software works with every modern and fast PC, and no further hardware is necessary.

### 3D Analysis

To compare different three-dimensional scans, reproducible and constant landmarks whose positions are not affected by weight loss were defined. The claviculae could be identified in each participant and were used to match different scans (Fig. 1b). Data were aligned using Geomagic Control 2014 Software (3D Systems Corporation, Rock Hill, SC, USA) using the built-in best fit algorithm. The volume of the torso was measured between the plane of both claviculae and the lowest edge of the abdomen with Comparison v3.40 (3D-Shape, Erlangen, Germany) (Fig. 1b). According to % total weight loss (%TWL), % total volume loss (%TVL) was calculated using Comparison v3.40 (3D-Shape, Erlangen, Germany) to describe the volume loss after surgery. For objective and reproducible two-dimensional measurements, two lines were defined to determine the circumference of the torso. The circumference at the umbilicus level (circumference umbilicus, *c*_umb_) was measured along the umbilicus and the most lateral part of the abdomen on both sides (Fig. 1b). The maximal abdominal circumference (circumference maximal, *c*_max_) was defined as the maximal distance along the widest lateral extend of the abdomen (Fig. 1b). These geodesic measurements were performed using Artec Studio 11.0 (Artec Group, Luxembourg, Luxembourg). To further describe the effect of weight loss on excess skin and sagging skin folds, distances between the center of the right clavicula and umbilicus (distance umbilicus, *d*_umb_) and the clavicula and the lowest point of the abdominal sagging skin fold (distance excess skin, *d*_es_) were calculated (Fig. 1b). To describe the operator and measurement error, the raw data of five three-dimensional scans were processed by two independent individuals. These individuals removed the head, arms, and legs at the described levels and measured the volume of the torsos.

### Statistical Analysis

Prism (version 7.0, GraphPad Software, La Jolla, CA, USA) was used to perform the statistical analyses and generate graphical representations of the data. The results are presented as the mean ± standard error of the mean unless otherwise specified. The distribution of variables was analyzed using the D’Agostino and Pearson normality test. All data were normal distributed and the significance of the differences between groups was determined by paired Student’s *t* tests. *p* value < 0.05 was considered statistically significant. Correlation was assessed by Pearson’s correlation coefficient or Spearman’s correlation coefficient.

## Results

### Population Cohort

The patient characteristics are detailed in Table 1. The mean preoperative body weight of the subjects was 157 kg, and the mean body mass index (BMI) was 53.3 kg/m^2^. Fifty-two percent of all procedures were sleeve gastrectomies (*n* = 13) and the others Roux-en-Y gastric bypasses (RYGB) (*n* = 12). The 6-month follow-up is also depicted in Table 1 and shows the measured weight loss.

**Table 1:** Patients’ characteristics

Characteristics	Included (*n* = 25)
Baseline	
Age (years)	45.08 ± 11.09
Male	9 (36)
ASA	2.52 ± 0.5859
Type 2 diabetes (%)	13 (52)
HbA1c all (mmol/mol)	45.74 ± 14.14
HbA1c diabetes (mmol/mol)	54.38 ± 15.8
SG	13 (52)
RYGB	12 (48)
Height (m)	1.716 ± 0.0755
Weight (kg)	157.1 ± 29.89
BMI (kg/m^2^)	53.34 ± 9.426
Waist circumference (cm)	149.2 ± 17.04
6-month follow-up	
Weight (kg)	120.5 ± 24.19
BMI (kg/m^2^)	40.91 ± 7.776
Type 2 diabetes (%)	7 (28)
HbA1c all (mmol/mol)	37.52 ± 9.113
HbA1c diabetes (mmol/mol)	42.89 ± 10.32
∆ BMI (kg/m^2^)	12.43 ± 3.99
%TWL	23.4 (12.17–33.83)
%EWL	47.6 (20.71–67.69)
Waist circumference (cm)	127.2 ± 14.97
%TVL	14.44 ± 6.658

Results are shown as the mean ± standard deviation or median (min-max) or absolute frequencies and rates (%)

### 2D and 3D Imaging of the Torso in Bariatric Patients Using Artec EVA

**Fig. 2:**
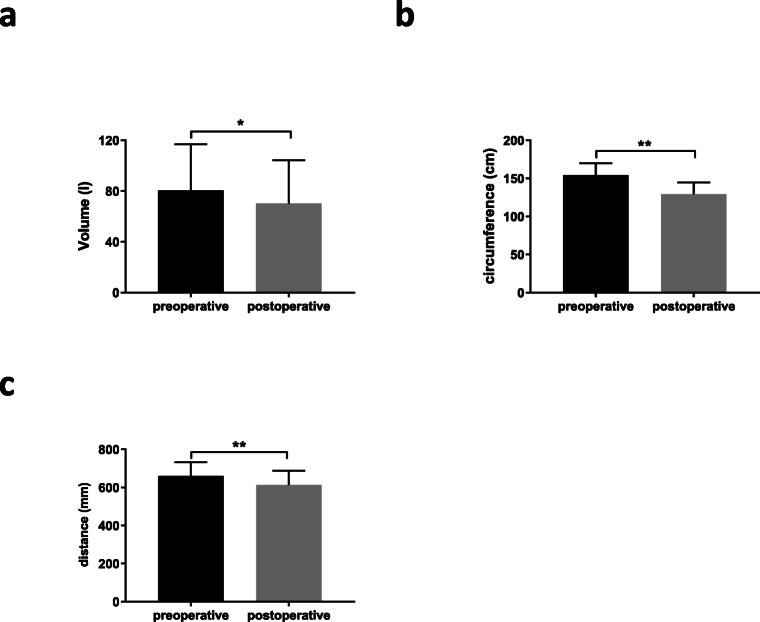
Pre- and postoperative measurements. **a** Pre- and postoperative volume of the torso. Comparison of pre- and postoperative **b***c*_umb_ and **c***d*_umb_. *p < 0.001, **p < 0.0001

Regardless of the high body mass of the bariatric patients, 2D and 3D imaging of the torso using Artec EVA® before and after bariatric procedures is possible. The claviculae were identified as consistent landmarks to match different scans in obese patients, especially after massive weight loss. Examples of three-dimensional reconstructions of the torso with subsequent quantification of volume or circumference loss are shown in Fig. 1. Heat maps of two matched scans illustrate the areas of body shape changes after bariatric surgery (Fig. 1d). Six months after surgery, the mean volume loss of the torso was 10.346 l (*p* < 0.0001) (Fig. 2a). Circumference loss (*c*_umb_ 24.76 ± 8.479, *p* < 0.0001; *c*_max_ 25.27 ± 13.12, *p* < 0.0001) and loss of distance (*d*_umb_ 38.11 ± 26.36, *p* < 0.0001 and *d*_es_ 47.33 ± 24.25, *p* < 0.0001) were also significant at the 6-month time point (Table 2 and Fig. 2b, c). The median measurement error between two independent operators was 0.031% (0.003–0.07%).

**Table 2:** 3D imaging studies

	Volume	Circumference		Distance	
	Torso (l)	Umbilicus (cm)	Abdominal excess skin (cm)	Umbilicus (mm)	Abdominal excess skin (mm)
Preoperative	80.52 ± 36.41	154 ± 15.96	158.8 ± 18.36	534.9 ± 56.7	659.6 ± 72.62
Postoperative	70.18 ± 34.03	129.3 ± 15.36	133.5 ± 14.63	496.8 ± 58.21	612.3 ± 76.22
∆	10.346 ± 4.720	24.76 ± 8.479	25.27 ± 13.12	38.11 ± 26.36	47.33 ± 24.25
*P*	< 0.0001	< 0.0001	< 0.0001	< 0.0001	< 0.0001

Results are shown as the mean ± standard error of the mean

### Correlation of Artec EVA® Scans with Clinical Parameters

**Fig. 3:**
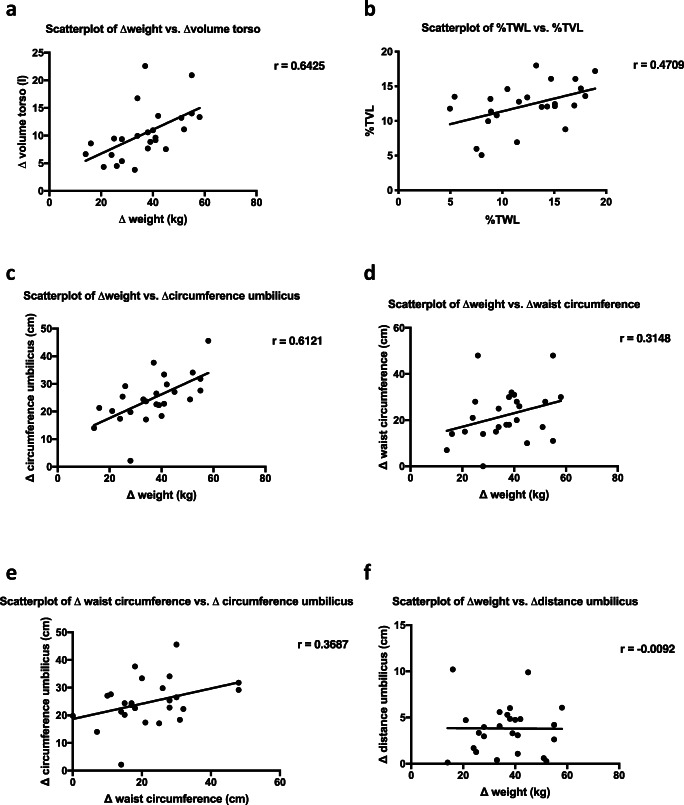
Correlation of Artec EVA® scans with clinical parameters. Scatterplots of **a** body weight loss vs. volume loss (*r* = 0.6425, *p* = 0.0005), **b** %TWL vs. %TVL (*r* = 0.4709, *p* = 0.0175), **c** body weight loss vs. *c*_umb_ (*r* = 0.6121, *p* = 0.0011), **d** body weight loss vs. change in waist circumference (*r* = 0.3148, *p* = 0.1254), **e** change in waist circumference vs. *c*_umb_ (*r* = 0.3687, *p* = 0.0697), and **f***d*_umb_ vs. body weight loss after bariatric surgery (*r* = − 0.0092, *p* = 0.9653)

There was a strong, positive monotonic correlation between body weight loss and volume loss after bariatric surgery (*r* = 0.6425, *p* = 0.0005, Fig. 3a). A moderate, positive correlation between %TWL and %TVL was found (*r* = 0.4709, *p* = 0.0175, Fig. 3b). Similar to the three-dimensional measurements, the circumferential measurement *c*_umb_ also correlated strongly with weight loss (*r* = 0.6121, *p* = 0.0011, Fig. 3c). In contrast to *c*_umb_, the change in the clinical measurement of waist circumference did not correlate significantly with weight loss after bariatric surgery (Fig. 3d). In line with this, we could not show a statistically significant correlation between the change in *c*_umb_ and waist circumference measured with a tape either (Fig. 3e). In addition, the changes in the distance between umbilicus and claviculae did not correlate with weight loss (Fig. 3f).

## Discussion

As obesity has become a worldwide epidemic, body shape and body shape changes have become increasingly important for the monitoring of therapy options such as the therapeutic effects of bariatric surgery. Due to several shortcomings of clinical measurements made with measuring tapes, three-dimensional techniques are on the rise. Artec EVA® is an easy-to-use bedside tool. The device is connected to a conventional notebook and can be used after a short briefing without extra equipment. The Artec EVA® system is capable of performing 2D and 3D imaging of the torso in bariatric patients. It is particularly important to visualize and compare pre- and postoperative changes in the body shape of bariatric patients. Therefore, it is crucial to match pre- and postoperative scans. For clinical measurements, this is particularly difficult in obese patients due to hidden anatomic landmarks. Therefore, in our study, participants stood straight with their feet close together and arms crossed behind their back, exposing the claviculae, which could then be reliably identified in all patients. Consequently, the claviculae were used as consistent landmarks to match different scans in obese patients, especially following bariatric surgery.

For the first time, a three-dimensional, bedside measurement of body shape changes in bariatric patients, as it is common in maxillofacial or vascular surgery [11–13] is possible. Mean volume loss of the torso was more than 10 l with 14.44 ± 6.658 %TVL 6 months after surgery. In particular, our three-dimensional method allows researchers to evaluate the entire torso independently of the rest of the body. Our data show that %TWL and % TVL (Fig. 3b) correlate. Nevertheless, we observed a difference between %TWL and %TVL, probably due to the fact that %TWL includes the weight loss of the entire body instead of only the torso. Therefore, focusing on the torso with a three-dimensional procedure may be of relevance for future studies addressing central obesity.

In addition to the better information provided by the third dimension, this system offers objective and reproducible measurements. The median interoperator variability of volume measurements of the torso was 0.03% in our study. This is an indicator for superior reliability and objectivity of our three-dimensional measurement technique. The reliability of manual measurement of waist circumference, which is widely used to describe central obesity, has been questioned in several studies [14–16]. In our study, we could demonstrate that there is no relevant correlation between the manual measurement of waist circumference with a measuring tape and weight loss (Fig. 3d) or *c*_umb_ (Fig. 3e). Especially in obese patients, it is nearly impossible to measure waist circumference with a measuring tape. It is difficult for a nurse or technician to place a tape around an obese person in a reliable manner. In contrast, postprocessing of three-dimensional data allows the objective and reproducible measurement of circumference (i.e., *c*_umb_ or *c*_max_) and any kind of distances (i.e., *d*_umb_ or *d*_max_). Consequently, *c*_umb_ correlated strongly with weight loss (Fig. 3c). Furthermore, hanging fat pads are not detected by two-dimensional measurement methods. This can lead to underestimation of the waist circumference in obese patients [17]. These shortcomings are overcome by volume measurements and the calculation of %TVL. Recent literature comparing three-dimensional techniques with anthropometric measurements of central obesity reports that three-dimensional scanners are a more reliable and reproducible method for detecting central obesity [18]. So far, we do not have an algorithm that can automatically distinguish between central obesity and hanging aprons. But the combination of volume measurement and the exact calculation of circumference as well as the possibility of defining any kind of distances helps to distinguish between central obesity and hanging aprons.

The incidences of overweight and obesity increase globally, and bariatric surgery is still the most effective treatment option for obesity. Therefore, three-dimensional imaging should become a common tool to measure central obesity in bariatric patients.

This is particularly important as waist circumference is used in many scientific publications and scientifically applied scores [19, 20]. The FLI score, for example, is widely used as a predictor for NAFLD and metabolic syndrome [21, 22] and for evaluating the effect of different treatment methods [23]. However, the FLI is still under investigation, and researchers continue to adjust how it is calculated to further improve the reliability [24]. Since the FLI also contains waist circumference, it is at risk for the above-mentioned sources of error. Our three-dimensional method can overcome these shortcomings and might be useful for research issues, to minimize measurement errors in science.

The objective calculation of %TVL is not the only innovative application of three-dimensional measurements. Another interesting feature of three-dimensional imaging is depicting the exact area of body mass reduction. It is possible to visualize certain areas of the body, i.e., the breast or different parts of the abdomen, and compare them pre- and postoperatively. Heat maps created by matching different scans from the same patient can precisely capture the change in body shape (Fig. 1d). Different shades of color illustrate the extent of change in body shape in the respective areas. Comparing these changes in body shape with postoperative quality of life or metabolism after bariatric surgery might be future aspects of using three-dimensional imaging. Surprisingly, *D*_es_ and *D*_umb_ decreased following weight loss (Table 2). Usually we expect the skin to sag following weight loss, but our data indicate that the skin does not sag, only the volume of the fat apron is reduced. The excess skin has a smaller volume and therefore seems to hang lower, whereas objective measurements prove the opposite. Correspondingly, *d*_umb_ did not correlate with weight loss (Fig. 3f). This finding shows that objective studies of body shape changes are missing in current literature.

Further fields of the application of three-dimensional scanners are reconstructive and plastic surgery after massive weight loss following bariatric interventions. Three-dimensional imaging has a long tradition in plastic surgery [25], and a more precise planning of surgical procedures, as demonstrated by Liu et al. for breast asymmetry [26], plays an important role in counseling postbariatric patients. Three-dimensional imaging can help to demonstrate the expected changes possible with a certain procedure. This is particularly important because a negative body image is associated with less weight loss and more depressive symptoms in postbariatric patients [27]. Hence, body image should be taken seriously and be part of the outcome assessment in pre- and postbariatric patients.

A few limitations should be considered regarding this study. Firstly, the investment costs for Artec EVA® are expensive compared to a measuring tape. Furthermore, scanning with Artec EVA® (15–30 s) and postprocessing the data (10–15 min) take more time than measurements with a tape (AE comments, comment # 2). In this study, we only measured the volume of the torso. Weight and volume loss of the extremities and the gluteal area are not detected by this method. Future studies of our group will therefore include scans of the entire body.

## Conclusion

Here, we present for the first time a three-dimensional measurement of the torso as an objective and reproducible indicator for abdominal obesity to complement the measurement of BMI and body weight in bariatric surgery patients. Furthermore, it can be used to visualize body shape changes after surgery or other body weight interventions. This technique can also be applied for research issues, e.g., the correlation of clinical scores (e.g., FLI) with histological diagnoses.

## Electronic Supplementary Material

ESM 1 (PPTX 88 kb)
