# Supplementary figures and images for: 3D Optical Imaging as a New Tool for the Objective Evaluation of Body Shape Changes After Bariatric Surgery
Source: Obes Surg. 2020 Jan 21;30(5):1866–73. doi: 10.1007/s11695-020-04408-4 (PMC7242279; doi:10.1007/s11695-020-04408-4)

## Slide 1
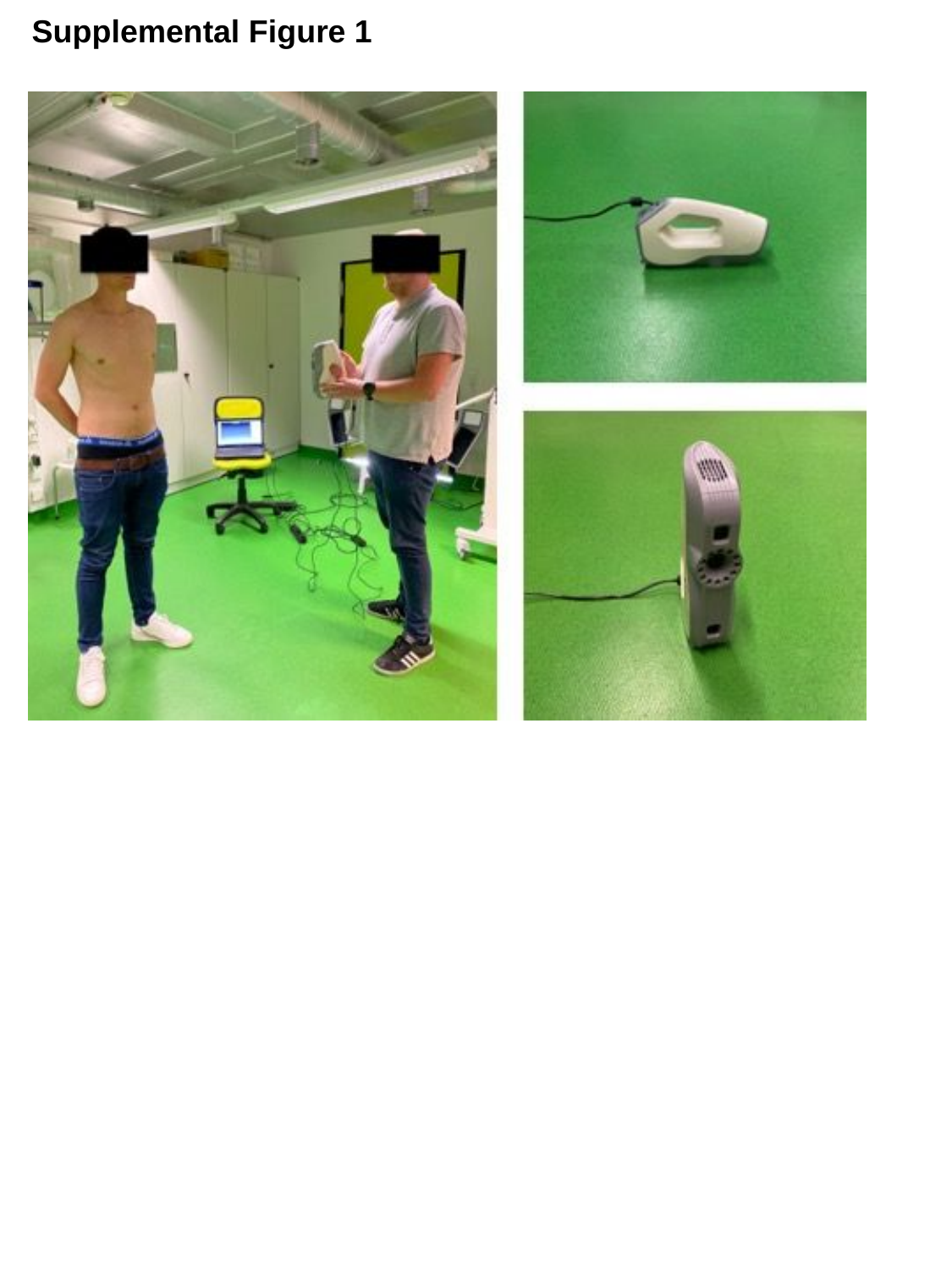

Supplemental Figure 1

Supplement: Supplementary file 1 — (PPTX 88 kb) [file 11695_2020_4408_MOESM1_ESM.pptx]
